# Causal relationship between endometrial cancer and risk of breast cancer: A 2-sample Mendelian randomization study

**DOI:** 10.1097/MD.0000000000038732

**Published:** 2024-06-28

**Authors:** Ye Liu, Lichao Zhu, Lei Guo, Jianhai Zhao, Jiang Li, Wenying Li, Ziyun Li, Shuai Chen, Jiapeng Zheng, Yating Zhao

**Affiliations:** a Affiliated Hospital of North China University of Science and Technology, Breast Disease Treatment Center, Tangshan, Hebei, China; b Tangshan Maternal and Child Health Centre, General Surgery, Tangshan, Hebei, China; c Affiliated Hospital of North China University of Science and Technology, General Surgery, Hebei, China.

**Keywords:** breast cancer, causal relationship, endometrial cancer, female, Mendelian randomization

## Abstract

Several studies have confirmed the important role of endometrial cancer (EC) in the development and progression of breast cancer (BC), and this study will explore the causal relationship between EC and BC by 2-sample Mendelian randomization analysis. Pooled data from published genome-wide association studies were used to assess the association between EC and BC risk in women using 5 methods, namely, inverse variance weighting (IVW), MR-Egger, weighted median (WME), simple multimaximetry (SM) and weighted multimaximetry (WM) with the EC-associated genetic loci as the instrumental variables (IV) and sensitivity analyses were used to assess the robustness of the results. The statistical results showed a causal association between EC and BC (IVW: OR = 1.07, 95% CI = 1.01–1.32, *P* = .02; MR-Egger: OR = 1.21, 95% CI = 0.71–1.51, *P* = .11; weighted median: OR = 1.05, 95% CI = 0.97–1.31, *P* = .19; simple plurality method: OR = 0.98, 95% CI = 0.81–1.15, *P* = .78; weighted plurality method: OR = 0.98, 95% CI = 0.81–1.14, *P* = .75), and the results of the sensitivity analyses showed that there was no significant heterogeneity or multiplicity, and the results were stable. EC is associated with an increased risk of developing BC. The results of this MR analysis can be used as a guideline for screening for BC in women with EC and to help raise awareness of screening for early detection and treatment.

## 1. Introduction

Endometrial cancer (EC) is the most common type of gynecological malignancy. Globally, the incidence of the disease is growing rapidly, with about 420,000 new cases in 2020.^[1]^ According to the general opinion, common risk factors for breast cancer (BC) include obesity, diabetes, early menarche, nulliparity, late onset menopause, and advanced age,^[2,3]^ and there is still a great deal of uncertainty regarding other underlying diseases such as EC. It has been shown^[4]^ that aromatase is commonly elevated in BC and EC, which catalyzes the key and rate-limiting enzyme in the conversion of androgens to estrogens in organisms, and that estrogens and their metabolites cause hyperproliferation and neoplastic transformation of breast and endometrial cells through proliferation and DNA damage. And fibroblast growth factor 2 (FGF2) amplification and overexpression have also been observed in 5% to 10% of BC and EC,^[5–7]^ fibroblast growth factor and its receptor have been implicated in the origin of human cancers. However, observational studies cannot confirm causality and their results may be affected by potential limitations of observational studies, such as confounding, reverse causality and measurement error.^[8,9]^ Given the possible confounding factors and the lack of randomized controlled trials, it is not yet possible to identify a potential causal relationship between EC and BC, which has important implications for our deeper understanding of the etiology and prevention methods of BC. Although randomized controlled trials (RCTs) are considered the highest standard for establishing causality, they were not feasible in this study.

Mendelian randomization (MR), study is a method of assessing the causal relationship between various exposures and disease outcomes by using genetic variation as an instrumental variable (IV).^[10]^ This analysis makes causal inferences by using genetic variation as a proxy for modifiable risk factors or health outcomes.^[11]^ Since genes are randomly assigned from conception, genetic variation is largely independent of other factors.^[12]^ MR studies need to satisfy 3 basic assumptions^[13]^: the assumption of relevance: genetic variation is associated with risk factors; the assumption of independence: genetic variation is independent of any known or unknown confounders; the assumption of exclusionary restriction: genetic variation affects only risk factors through the risk factor results.

Two-sample Mendelian randomization involves 2 different study populations. For example, EC is measured in 1 sample and BC in the other. This design has 2 advantages: one, all MR studies do not need to collect risk factors and outcomes. Two, the results can be very large (usually > 50,000), allowing the use of pooled results from genome-wide association studies, and therefore the results are precise and statistically very efficient. It can also address challenges such as difficult or costly sample collection.^[14]^

In this study, we used MR methods to explore the potential causal relationship between EC and BC-related genetic variants.

## 2. Materials & methods

### 2.1. Data sources for MR analysis

EC was derived from the genome-wide association study (GWAS) pooled data, including 121,885 European individuals, which included a total of 121,885 European individuals, comprising a total of 12,906 patients and 108,979 controls, with 9470,555 single nucleotide polymorphisms (SNPs) and the pooled data were publicly available from https://gwas.mrcieu.ac.uk/ datasets/ebi-a-gcst006464/. BC data were obtained from the GWAS (iCGOS) pooled data, which included a total of 89,677 European individuals, comprising a total of 46,785 BC patients and 42,892 controls, and the pooled GWAS results included 10,680,257 SNPs, https://gwas.mrcieu.ac.uk/ datasets/ ieu-a-1130/, and all participants were European females.

This study was a reevaluation of existing and publicly available data; therefore, no ethical authorization was necessary. Information on the exposure factor EC and outcome BC samples used in this study is detailed in Table 1.

**Table 1:** Details of samples included in MR study.

Variant	Sample size (n)	Case group (n)	Control subjects (n)	Place of origin of the sample	Yr of publication	PubMed ID	Website
Endometrial cancer (EC)	121885	12906	108979	European	2018	30093612	https://gwas.mrcieu.ac.uk/datasets/ebi-a-GCST006464/
Breast cancer (BC)	89677	46785	42892	European	2017	29059683	https://gwas.mrcieu.ac.uk/datasets/ieu-a-1130/

MR = Mendelian randomization.

### 2.2. Study design

**Figure 1.**
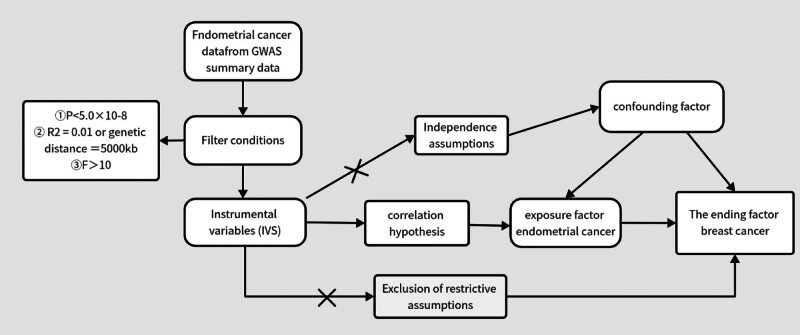
Schematic of MR core assumptions. MR = Mendelian randomization.

This study adopted a 2-sample Mendelian randomization approach to validate the causal relationship between the genetically predicted exposure factor EC and the outcome BC using EC as the exposure factor, genetic variant SNPs as IV and BC as the outcome factor. MR analysis needs to follow 3 core assumptions^[15]^: firstly, the assumption of associativity: SNPs of the IV must be strongly associated with exposure factors; second, the independence assumption: genetic variants are independent of confounders affecting exposure and outcome; and finally, the exclusivity assumption: SNPs can only have an effect on outcome through exposure. A schematic of Mendel core assumptions is shown in Figure 1.

### 2.3. Selection of IV

SNPs must satisfy the following conditions: *P* < 5.0 × 10^-8^, which indicates that they have a significant correlation with EC; set R^2^ = 0.01 or genetic distance = 5000kb, and remove SNPs with R^2^ > 0.01 in the range of 5000kb to the most significant SNPs, remove chained unbalanced balanced SNPs; remove SNPs with palindromic structure; and remove SNPs with F < 10 to exclude weak tools. Weak instruments are those genetic variants that have low potency to explain exposure and, although associated with the exposure factor, have little effect on the exposure factor and provide little statistical power to test the hypothesis. This can lead to problems such as imprecise estimates of causal effects and increased probability of Type I error.^[13]^ The strength of IV can be evaluated by the F statistic, which is calculated for individual SNPs as F = bate^2^/se^2,[16]^ and is less affected by weak instrumental bias when F > 10. Based on the GWAS results of EC and BC, we can co-collate SNPs with the same allele to make the effect values of exposure and outcome correspond to each other.^[17]^

### 2.4. Statistical treatment

In this study, the inverse variance weighted (IVW) method was used as the primary research method to assess the potential causal association between EC and BC. This method is best suited for situations where genotypes are highly balanced with exposure factors and can produce unbiased estimates. In this study, the MR-Egger intercept test was used to assess the presence of horizontal pleiotropy, and if *P* > .05, horizontal pleiotropy did not exist.^[18]^ In addition, the study performed leave-one-out analysis to assess the effect of individual SNPs on significant effects. Cochran Q-test was used to detect the heterogeneity situation; if *P* < .05, heterogeneity exists, and at this point, one should choose to apply the random-effects IVW model.^[19]^ leave-one-out method was used to conduct sensitivity analyses, and after deleting 1 SNPs in turn one by one, the IVW method was reapplied to estimate the causal effects of the remaining SNPs. This method was used to assess whether the MR causal estimation results might be affected by SNPs with a particularly large level of multiplicity effect, according to which the robustness of the MR causal estimation results was judged. However, the IVW method may lead to bias when there is multiplicity or imbalance in the data. Therefore, it was supplemented with MR-Egger method, weighted median (WME), simple mode (SM) and weighted mode (WM).

The above methods were performed using the R software (version 4.3.3) with the R package “TwoSampleMR (version 0.5.6)” for statistical analysis and data visualization. Since the IVW method is more efficient than the other 4 MR methods, the IVW method was used to test the causal effect in this study. Due to the relaxation of the threshold requirement, in order to avoid increasing the risk of 1 type of error, and to verify whether the results of the study are affected by multiple testing.

## 3. Results

### 3.1. Determination of IV

SNPs loci with gene-wide significant (*P* < .05) association with EC were selected for pooling using R software, and a total of 13 SNPs were included as IV by excluding the control step of chain disequilibrium interference. As a single SNP of IV, the range of F statistic was 30.37 to 84.82, indicating that there was a strong correlation between SNPs and exposure, and the results of MR analyses were unlikely to be biased by the influence of weak IV (see Table 2).

**Table 2:** List of SNPs screened for factors exposed in this study.

SNP	chr	EA	OA	EC			BC			F
				*beta*	*SE*	*P*	*beta*	*SE*	*P*	
rs10089519	8	G	A	0.09	0.02	3.02 × 10^-8^	3.87 × 10^-3^	0.01	.70	30.70
rs10835920	11	T	C	0.09	0.02	1.33 × 10^-8^	4.89 × 10^-3^	0.01	.63	32.29
rs113998067	1	C	T	0.20	0.04	3.58 × 10^-8^	3.17 × 10^-3^	0.02	.20	30.37
rs11651052	17	G	A	0.14	0.02	4.00 × 10^-20^	1.83 × 10^-3^	0.01	.06	84.42
rs148261157	2	A	G	0.23	0.04	3.39 × 10^-8^	9.21 × 10^-3^	0.03	.78	30.47
rs1679014	9	C	T	0.17	0.03	6.38 × 10^-9^	9.65 × 10^-3^	0.02	.62	33.72
rs1740828	6	A	G	0.14	0.02	4.15 × 10^-16^	8.20 × 10^-4^	0.01	.95	66.16
rs17601876	15	G	A	0.12	0.02	3.27 × 10^-14^	2.33 × 10^-2^	0.01	.02	57.56
rs2747716	6	G	A	0.10	0.02	2.91 × 10^-10^	6.74 × 10^-3^	0.01	.49	39.73
rs4733613	8	G	C	0.15	0.02	3.11 × 10^-12^	8.08 × 10^-3^	0.01	.58	48.62
rs7981863	13	T	C	0.15	0.02	2.70 × 10^-17^	2.98 × 10^-2^	0.01	.01	71.55
rs882380	17	A	C	0.10	0.02	4.66 × 10^-9^	2.01 × 10^-3^	0.01	.84	34.33
rs937213	15	C	T	0.09	0.02	5.07 × 10^-9^	3.00 × 10^-4^	0.01	.98	34.16

beta = allele effect value, chr = chromosome, EA (effect allele) = effect allele, OA (other allele) = non-effect allele, SE = standard error, SNP = single nucleotide polymorphism.

### 3.2. Causal relationship between EC and BC

**Figure 2.**
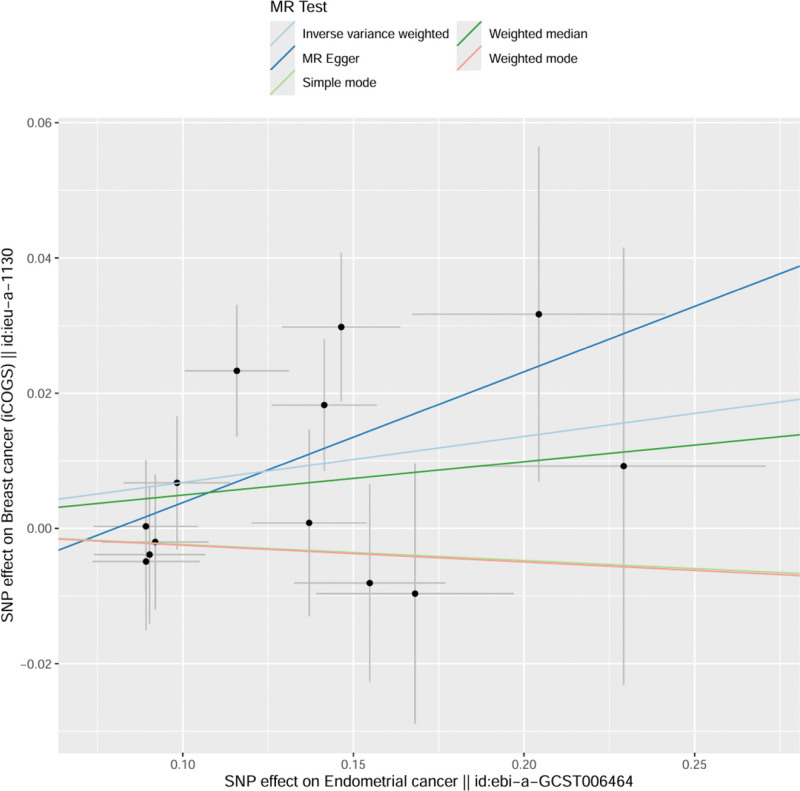
Scatter plot of EC and BC. BC = breast cancer, EC = endometrial cancer.

The results of MR analysis showed that IVW: OR = 1.07, 95% CI = 1.01–1.32, *P* = .02; MR-Egger: OR = 1.21, 95% CI = 0.71–1.51, *P* = .11; WME: OR = 1.05, 95% CI = 0.97–1.31, *P* = .19; Simple mode: OR = 0.98, 95% CI = 0.81–1.15, *P* = .78; Weight mode: OR = 0.98, 95% CI = 0.81–1.14, *P* = .75. The direction of causality obtained by the 5 methods was the same, showing that patients with EC increased the risk of BC, and the IVW showed a statistically significant difference (*P* < .05) (see Table 3 and Fig. 2).

**Table 3:** Results of MR analysis of the relationship between EC and BC.

Exposure factor	Methodologies	OR	95% CI	*P*
Endometrial cancer (EC)	MR-Egger	1.21	0.97–1.51	.11
	WME	1.05	0.97–1.31	.19
	IVW	1.07	1.01–1.32	.02
	Simple mode	0.98	0.83–1.15	.78
	Weighted mode	0.98	0.83–1.14	.75

CI = confidence interval, IVW = inverse variance weighting method, MR = Mendelian randomization, OR = dominance ratio, WME = weighted median method.

### 3.3. Sensitivity analysis

Cochrane test showed that heterogeneity existed among IV if *P* < .01. The heterogeneity result of IVW was *P* = .34, and the heterogeneity result of MR-Egger regression was *P* = 0. 36, suggesting that there was no heterogeneity among the 13 SNPs. See Table 4. MR-Egger regression analysis intercept *P* > .05, thus indicating that there is little risk of potential confounding bias in the analysis results.

**Table 4:** Two-sample Mendelian randomization analysis SNP tool variable information table.

Exposure	Heterogeneity test		Multiplicity test	
	MR-Egger Q *P* value	IVW Q *P* value	MR-Egger intercept value	MR-Egger intercept *P* value
EC	0.36	0.34	−0.02	0.27

MR = Mendelian randomization, Q = Cochran Q-value.

**Figure 3.**
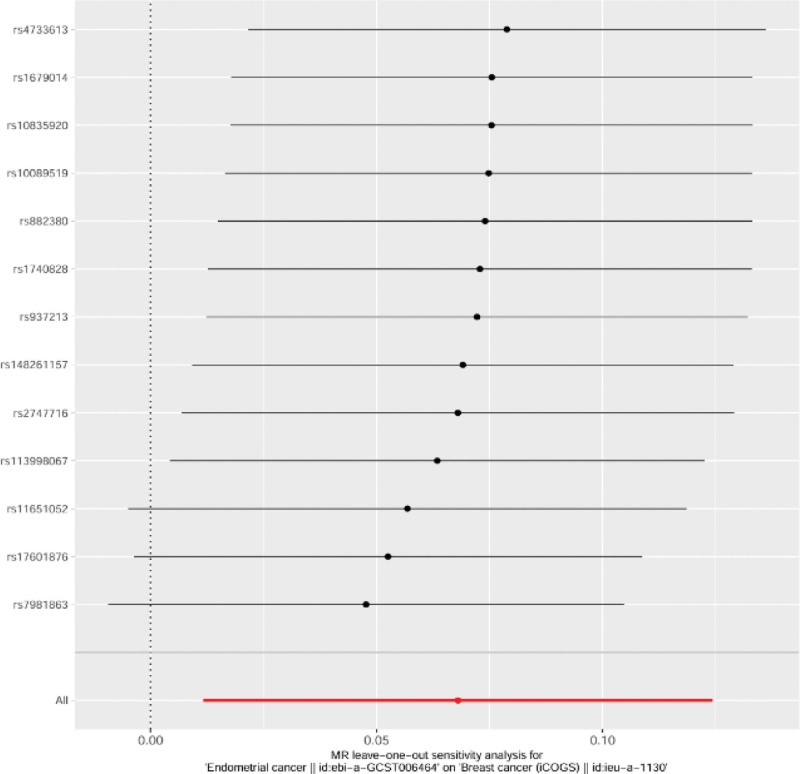
Analysis of EC and BC by the leave-one-out method. BC = breast cancer, EC = endometrial cancer.

The results of leave-one-out sensitivity analysis showed that after the stepwise exclusion of 12 SNPs. The remaining 13 SNPs were similar to the combined ORs of the IVW method (*P* > .05), and no SNPs were found to be present in IV that had an impact on the results (see Fig. 3), suggesting that the effect ORs derived from the IVW method were robust and that the results of the MR analyses were not driven by a single SNP.

## 4. Discussion

We conducted a 2-sample MR analysis using large-scale GWAS summary statistics in 12906 EC patients and 108,979 controls with 46785 BC patients and 42892 controls with the aim of exploring the putative causal relationship between BMI and EC risk. The causal relationship was further confirmed by the IVW method, MR-Egger regression analysis, WME method, SM and WM. The results of the sensitivity analysis of the leave-one-out method showed that the findings were not due to the abnormal effects of a single SNP, enhancing the reliability of the conclusions. Based on this study, we found a positive causal association between genetically predicted EC exposure and the risk of developing BC.

EC, as the second most common malignant tumor of the female reproductive system in China, is usually divided into 2 categories, with estrogen-sensitive EC accounting for 80% to 85% of all EC. There are 3 main pathways of estrogen production in EC patients,^[20,21]^ firstly, estradiol secreted by the ovaries reaches the EC tissues through the somatic circulation. Secondly, aromatase enzymes present in adipose tissue and skin catalyze the conversion of circulating androstenedione to estrone (E1) and then to E2, both of which enter the circulation and reach the endometrium. The last source is the synthesis of estrogen in EC tissue.

Both BC and EC are hormone-dependent malignant tumors with estrogen as a common risk factor, especially breast tissues are exposed to significantly increased levels of estrogen.^[22]^ Guo et al^[23]^ found that in the EC population, the incidence of BC was 4 to 8 times higher than in the general population. Estrogen is tissue-specific and its receptors are widely distributed in breast and endometrial tissues, and there is a local increase of estrogen in BC and EC tissues.^[24,25]^ In addition, some studies have revealed a tendency for EC patients to be older, which implies the age distribution characteristics of patients with recurrent EC and BC, which may explain the increased incidence of BC, that is, the accumulation of mutations in the genetic material.^[23]^ Tumor generation and progression is a slow and dynamic process, and during continuous growth, tumor cells undergo genetic mutations that lead to evolution toward more aggressive tumor cells.^[26]^ BRCA-1,^[27]^ BRCA-2,^[27]^ P53,^[28,29]^ HER-2^[30]^ and PTEN,^[31]^ as susceptibility genes for EC, are equally susceptible to BC.

This 2-sample MR randomization study is of extreme clinical importance. Firstly, as the first MR study to use EC as an exposure to estimate its causal effect on BC risk. A major strength of this study is its 2-sample MR design, which prevents the influence of potential confounders. Second, the study sample was obtained from the GWAS database,^[32]^ making the study a valid causal inference with high statistical power. Third, the results of the study were extremely robust through strict quality control conditions and a series of sensitivity analyses. After removing SNPs associated with potential confounders, the MR study demonstrated a causal relationship between genetically predicted EC and the risk of BC, with independent associations between the 2.

However, there are some limitations to this study. First, the SNPs in the study were predominantly from European populations, so our causal estimates may not be fully generalizable to other populations. Second, as previous studies have assessed the causal relationship between factors such as weight and BMI and EC, as well as their causal relationship with BC, and found them to be causally associated with both EC and BC risk.^[2,33]^ Based on this, we did not conduct the study again. Third, due to a lack of information, it was not possible to stratify the outcomes according to different clinical subtypes of BC, which is important in clinical practice. Subsequent further studies on BC with different clinical subtypes are needed to fully explore the association between EC and BC.

## 5. Conclusion

In conclusion, in this study, the positive causal relationship between exposure to EC and outcome BC was explored, but the mechanisms and details need to be discovered by further research, with a view to clinical practitioners for patients with EC, to strengthen the screening of BC, to achieve early detection, early diagnosis and early treatment.

## Acknowledgments

All genetic summary data were obtained from the IEU OpenGWAS project. We thank all participants and investigators for contributing to the GWAS data.

## Author contributions

**Conceptualization:** Ziyun Li.

**Data curation:** Ye Liu.

**Formal analysis:** Ye Liu, Jiapeng Zheng.

**Funding acquisition:** Yating Zhao.

**Investigation:** Lei Guo, Jiang Li.

**Methodology:** Ye Liu.

**Project administration:** Shuai Chen.

**Software:** Jiang Li.

**Supervision:** Jianhai Zhao, Wenying Li.

**Validation:** Lichao Zhu.

**Visualization:** Lichao Zhu.

**Writing – original draft:** Ye Liu.

**Writing – review & editing:** Yating Zhao.
